# Blindness as an Initial Presentation of Rectal Cancer with Brain Metastases

**DOI:** 10.4103/1319-3767.45065

**Published:** 2009-01

**Authors:** Tejinder Singh, K. V. Sajeevan, Ankit Jain, Ullas Batra, K. S. Saini, C. T. Satheesh, K. C. Lakshmaiah, K. Govind Babu, D. Lokanatha

**Affiliations:** Department of Medical Oncology, Kidwai Memorial Institute of Oncology, Bangalore-560 030, India. E-mail: drtejinderseera@rediffmail.com

Sir,

Brain metastases frequently occur after the diagnosis of systemic cancer. However, some patients with brain metastasis may be diagnosed before the primary tumor is found.[1] Colorectal cancer patients with brain metastasis have a poor prognosis because of the tendency of these patients to have a higher frequency of cerebellar metastases. Patients with brain metastasis as the only manifestation of an undetected primary tumor have a favorable prognosis with an overall median survival of 13.4 months. Patients with colorectal carcinoma tend to have a poorer prognosis. This may be due to the tendency of these patients to have a higher frequency of cerebellar metastases, which are associated with an adverse prognosis.[2]

**Figure 1:**
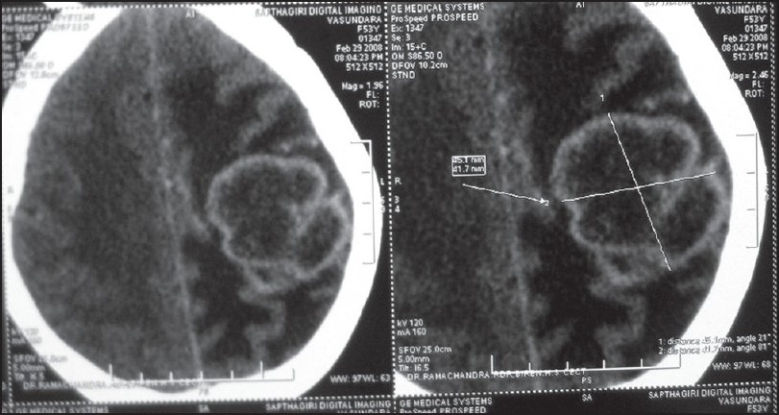
CT scan of the brain shows large left parietal space occupying lesion with perilesional edema

**Figure 2:**
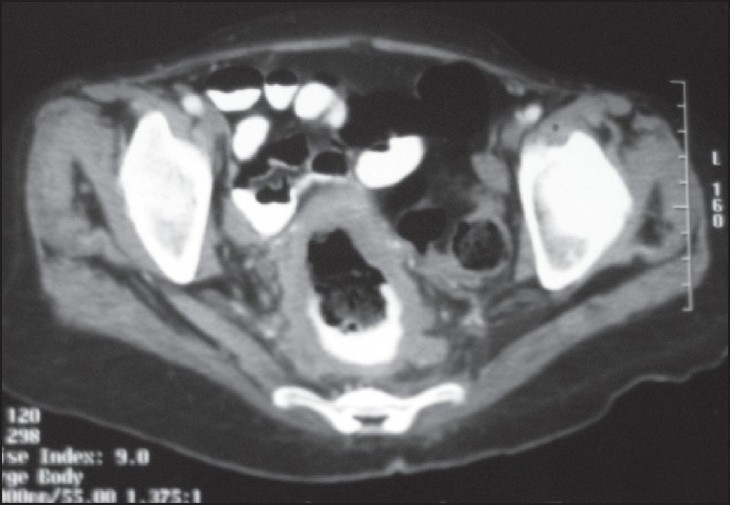
CT scan of the abdomen shows rectal mass with perirectal infiltration

**Figure 3:**
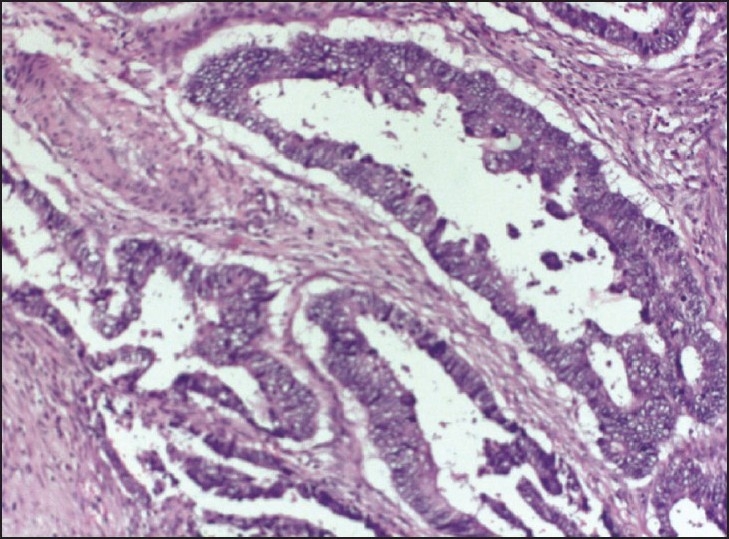
Neoplasm composed of pleomorphic epithelial cells arranged in glandular pattern (H and E;×40)

A 55-year-old woman presented with sudden-onset bilateral blindness and altered sensorium of 15 days duration. On ophthalmologic examination, both pupils were normal and direct and consensual reflexes were present. Fundoscopy revealed bilateral optic atrophy secondary to papilloedema. computed tomography (CT) scan of the brain showed a large lesion occupying left parietal space with perilesional edema [Figure 1]. Stereotactic biopsy of the brain lesion revealed metastatic adenocarcinoma. On further workup, the CT scan of the abdomen showed irregular circumferential wall thickening of the rectum with perirectal infiltration [Figure 2]. There were no metastases in liver and lung. She was treated with steroids, mannitol, and palliative cranial radiotherapy of 30 Gy of 10 fractions. Once her condition stabilized, colonoscopic-guided biopsy of rectal mass was performed. Histopathological examination revealed adenocarcinoma of the rectum [Figure 3]. She was treated with 5 Fluorouracil-lecovorin regimen and tolerated chemotherapy well, and her vision has improved marginally. She did not receive concurrent radiotherapy as her general condition was poor. She has finished four cycles of chemotherapy and is doing well.

Metastatic brain mass is uncommon and frequently associated with metastatic disease elsewhere. Older estimates suggested that metastases constituted of 20–40%[1] of all intracranial tumors. In adults, the commonest sources of brain metastases are the lung, breast, gastrointestinal tract, genitourinary tract, and malignant melanoma. The best diagnostic test for brain metastases are contrast-enhanced magnetic resonance imaging (MRI) and CT scan. Other diagnostic measures being needed to establish the diagnosis firmly are angiog raphy and/or biopsy. Stereotactic biopsy has become the most convenient and safe approach to obtain a tissue diagnosis.[3] Untreated brain metastases are associated with a median survival time of about 4 weeks and poor prognosis.[4] At the time of diagnosis of colorectal carcinoma, 2–3% of patients are likely to be harboring brain metastases, and another 10% of patients will develop brain lesions during the course of their disease. Surgical removal of colorectal metastatic brain lesions in selected cases may result in a longer survival time. Increased awareness of the possibility of brain metastases, early diagnosis, and aggressive therapy can increase the survival of patients with colorectal cancer with brain metastases.[2]
